# Supplementary material for: Assessing the Psychometric Properties of the Illness Management and Recovery Scale: A Systematic Review Using the Consensus-Based Standards for the Selection of Health Measurement Instruments (COSMIN)
Source: Behav Sci (Basel). 2024 Apr 18;14(4):340. doi: 10.3390/bs14040340 (PMC11047639; doi:10.3390/bs14040340)
Supplement: Supplementary file 1 [file behavsci-14-00340-s001.zip › behavsci-2926757-supplementary.pdf]

Table S1. Methodological quality of assessments of the measurement properties of the IMR-s.

Article	Box 2	Box 3	Box 4	Box 6	Box 7	Box 9	Box 10
[21]	NR	NR	NR	NR	NR	Adequate	NR
[33]	NR	NR	NR	NR	NR	NR	Doubtful
[37]	NR	NR	NR	NR	NR	NR	Inadequate
[59]	NR	NR	NR	NR	Adequate	Adequate	Inadequate
[4]	NR	NR	NR	NR	NR	NR	Inadequate
[28]	NR	NR	NR	NR	NR	NR	Doubtful
[29]	NR	NR	NR	NR	NR	NR	Inadequate
[67]	NR	NR	NR	NR	NR	NR	Inadequate
[31]	NR	NR	Very good	NR	NR	NR	Doubtful
[66]	NR	NR	NR	NR	NR	NR	Inadequate
[18]	NR	NR	Very good	NR	NR	NR	Doubtful
[20]	NR	NR	NR	NR	NR	NR	Doubtful
[32]	NR	NR	Very good	Adequate	NR	Very good	NR
[35]	NR	NR	NR	NR	NR	NR	Inadequate
[34]	NR	NR	NR	NR	NR	NR	Inadequate
[36]	NR	NR	Very good	NR	NR	Very good	NR
[25]	NR	NR	NR	NR	NR	NR	Inadequate
[45]	NR	NR	NR	NR	NR	NR	Doubtful
[38]	NR	NR	Very good	NR	NR	NR	Doubtful
[39]	NR	NR	Very good	NR	NR	NR	Iandequate
[41]	NR	NR	NR	NR	NR	NR	Inadequate
[30]	NR	NR	Very good	Adequate	NR	Very good	NR
[15]	NR	Adequate	Very good	NR	NR	Adequate	NR
[50]	NR	NR	Very good	NR	NR	NR	Inadequate
[42]	NR	NR	NR	NR	NR	NR	Doubtful
[44]	NR	NR	NR	NR	NR	Very good	NR
[16]	NR	NR	NR	NR	NR	NR	Doubtful
[43]	NR	NR	Very good	NR	NR	Doubtful	NR
[47]	NR	NR	NR	NR	NR	Doubtful	NR

Table S1. Methodological quality of assessments of the measurement properties of the IMR-s.

[46]	NR	NR	Very good	NR	NR	Very good	NR
[53]	NR	Very good	Doubtful	NR	NR	NR	NR
[49]	NR	NR	Very good	NR	NR	NR	Inadequate
[51]	NR	NR	NR	NR	NR	NR	Inadequate
[6]	NR	NR	Very good	NR	NR	NR	Inadequate
[27]	Doubtful	NR	Very good	Adequate	NR	NR	NR
[52]	NR	NR	Very good	NR	NR	NR	Doubtful
[54]	NR	NR	NR	NR	NR	NR	Inadequate
[19]	NR	NR	NR	NR	NR	NR	Inadequate
[3]	NR	NR	NR	NR	NR	NR	Inadequate
[55]	NR	NR	NR	NR	NR	Adequate	NR
[56]	NR	NR	Very good	Adequate	NR	Very good	NR
[57]	NR	NR	NR	NR	NR	NR	Inadequate
[17]	NR	NR	NR	NR	NR	NR	Inadequate
[26]	NR	Very good	Very good	NR	NR	Very good	NR
[40]	NR	NR	Doubtful	NR	NR	NR	Doubtful
[58]	NR	NR	Very good	NR	NR	Very good	NR
